# Visual Rewards From Observation for Sequential Tasks: Autonomous Pile Loading

**DOI:** 10.3389/frobt.2022.838059

**Published:** 2022-05-31

**Authors:** Nataliya Strokina, Wenyan Yang, Joni Pajarinen, Nikolay Serbenyuk, Joni Kämäräinen, Reza Ghabcheloo

**Affiliations:** ^1^ Computing Sciences, Tampere University, Tampere, Finland; ^2^ Department of Electrical Engineering and Automation, Aalto University, Espoo, Finland; ^3^ Automation Technology and Mechanical Engineering, Tampere University, Tampere, Finland

**Keywords:** visual rewards, learning from demonstration, reinforcement learning, field robotics, earth moving, visual representations

## Abstract

One of the key challenges in implementing reinforcement learning methods for real-world robotic applications is the design of a suitable reward function. In field robotics, the absence of abundant datasets, limited training time, and high variation of environmental conditions complicate the task further. In this paper, we review reward learning techniques together with visual representations commonly used in current state-of-the-art works in robotics. We investigate a practical approach proposed in prior work to associate the reward with the stage of the progress in task completion based on visual observation. This approach was demonstrated in controlled laboratory conditions. We study its potential for a real-scale field application, autonomous pile loading, tested outdoors in three seasons: summer, autumn, and winter. In our framework, the cumulative reward combines the predictions about the process stage and the task completion (terminal stage). We use supervised classification methods to train prediction models and investigate the most common state-of-the-art visual representations. We use task-specific contrastive features for terminal stage prediction.

## 1 Introduction

**FIGURE 1:**
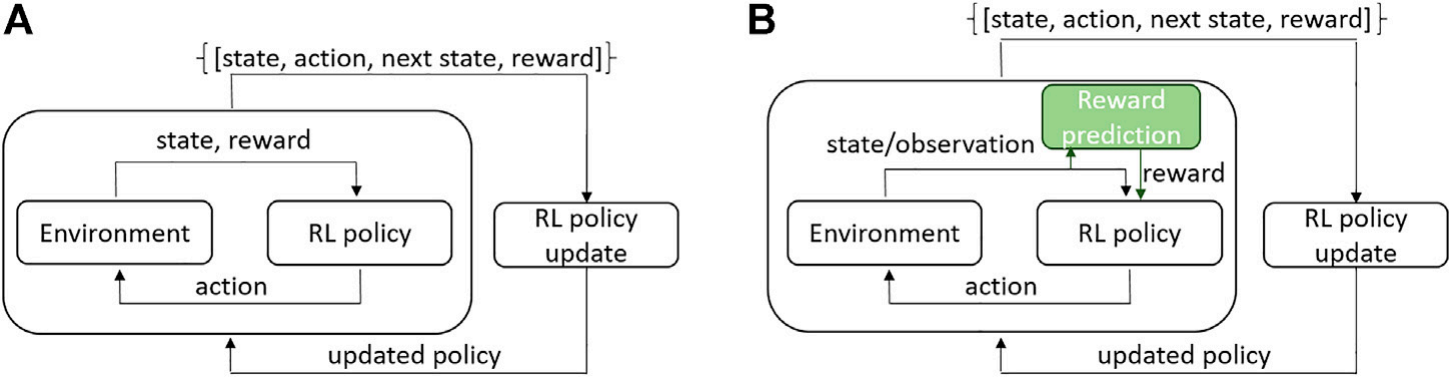
RL architecture: **(A)** classical online RL where state and reward are returned by the environment; **(B)** a real-world scenario where instead of full state only its observation is obtained and reward is predicted from the observation. Our contribution lies in the reward prediction block marked in green.

In classical Reinforcement Learning (RL) architecture (Sutton and Barto 2018), an agent acts upon an environment, receives feedback in form of reward, and observes the state of the environment (see Figure 1A). The collected experience is used to update the agent’s policy. The process repeats until the agent converges to the desired behavior. Reward encodes the task objective. For example, the closer the robot is to task completion the higher the reward is. RL has demonstrated impressive results in simulated environments (Kaiser et al., 2020; Yu and Rosendo 2021) and for robotic tasks in controlled laboratory conditions (Zhu et al., 2020); Koert et al., 2020; Veiga et al., 2020). In real-world large-scale applications, such as those found in field robotics, the full state of the environment cannot be received. Instead, the robot only obtains an observation of the environment state through the sensors (see Figure 1B). Moreover, the tasks usually require long-horizon decision-making and the reward function is difficult to engineer. In literature, reward learning is referred to as an inverse RL problem Osa et al. (2018). Learning the reward function online from interactions with the environment is challenging when the amount of training samples is limited and training time for the RL algorithm is restricted. The desirable solution would be to estimate the reward from environment observation using the prior collected experience, i.e., as learning from demonstration. In this paper, we address the problem of visual reward estimation for multi-stage robotic applications and study its reliability in significantly varying conditions. Our test case is the autonomous pile-loading task implemented on a real-scale robotic wheel-loader in changing outdoor weather conditions.

Pile loading is one of the most challenging tasks in earth moving automation for heavy-duty mobile machines. This is partly caused by the difficulty of modelling the interaction between the tool and the material (Dadhich et al., 2016) and partly because of high variation in worksites and weather conditions throughout the year. Weather conditions affect the material properties, the hydraulics properties of the machine, and the ground surface properties. The majority of the state-of-the-art works on pile loading or excavation automation are either model-based or use heuristics (Sotiropoulos and Asada 2020), and experimented in simulators or with toy setups. Therefore it is unclear how well these methods perform in real worksites. Recently several works implemented reinforcement learning based autonomous pile-loading (Azulay and Shapiro 2021; Backman et al., 2021), which were also demonstrated either on a toy or simulated set-up. Existing progress and our own experience (Yang et al., 2020; Yang et al., 2021) indicate complexity of this real-world problem. G. Dulac-Arnold and Mankowitz, (2019) reports nine challenges of real-world RL, among which sample efficiency, safety constraints, large or unknown delays in the system actuators, high-dimensional state and action spaces, etc. In this work, we focus on one of the challenges - reward learning. Specifically, we investigate vision-based reward estimation for a real-world set-up learned from demonstrations.

**FIGURE 2:**
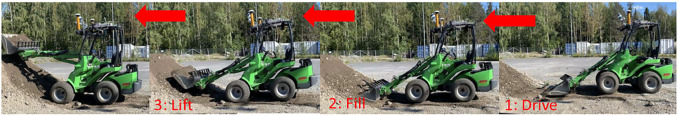
Example of the typical stages in the pile loading task.

**FIGURE 3:**
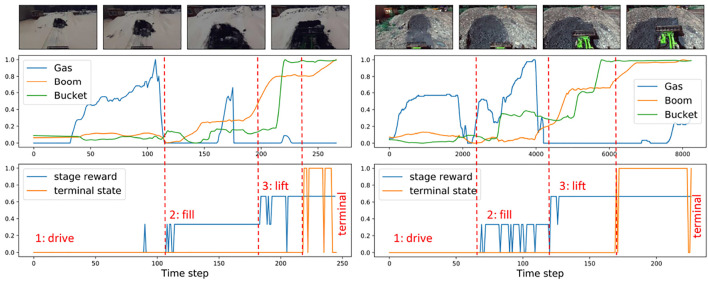
Demonstration examples and the predicted stages: visual observation (top), corresponding to them gas commands and joint angles (middle), predicted stages of progress (bottom).

Our experimental set-up is illustrated in Figure 2 where a robotic wheel-loader performs the task of loading a pile of material and lifting the boom up. The wheel loader is equipped with a stereo camera providing an egocentric view. This is a long-horizon task where a suitable reward function is hard to engineer even using expert knowledge. Several previous works suggest learning a reward together with the policy online (Ho and Ermon 2016; Fu et al., 2017; Ghasemipour et al., 2020). To the best of our knowledge, there is no demonstration of this method for the long-horizon task in highly varying real-world conditions. Moreover, having an initial approximation of the reward function is desirable in the long-horizon tasks. Sermanet et al. (2017b) proposed a stage-based visual reward estimation approach and demonstrated it on a door opening task in laboratory conditions. This approach is attractive for field robotics applications since it requires only minimum information from an expert about the stages of the task and initially can be learned offline. Figure 3 shows an example of such rewards for the pile-loading task. At each time step, a reward is associated with the stage of the task. Additionally, we study the sparse reward prediction based on the outcome of the task using task-specific visual features that previously demonstrated good performance in training a behavior cloning controller Yang et al. (2020). In our work, unlike in Sermanet et al. (2017b), we report results for several visual representations, including, time-contrastive representations, depth, and selected deep features. We propose that the intermediate stage and the sparse terminal stage rewards can be combined into cumulative reward.

To summarize, our work provides the following contributions:

• we review the methods of reward estimation and visual representations used in learning-based approaches for robotics applications; additionally, we overview the progress of learning-based methods in autonomous earth moving;
• we propose a framework where the cumulative reward combines two predictions from visual observation: the current stage of the progress and whether the task has been completed (terminal stage). We formulate the prediction as a supervised classification task and investigate the most common state-of-the-art visual representations. For the terminal stage prediction, we test task-specific visual features.
• the framework has been implemented and tested on an actual scale autonomous wheel-loader during three seasons (summer, autumn, and winter).

## 2 Related Work

*Reward learning–*Several methods propose learning the reward function by iteratively optimizing the reward and agent behavior while interacting with the environment, e.g., Ho and Ermon (2016); Fu et al. (2017); Ghasemipour et al. (2020). They are based on an adversarial paradigm as in Generative Adversarial Networks (GANs) (Goodfellow et al., 2014). The generator learns the policy and the discriminator learns to differentiate expert transitions from a non-expert. The reward is associated with a confusion of a discriminator. These methods minimize f-divergence between the expert and the learning agent state-action distributions Ghasemipour et al. (2019). With the generator trying to maximize the reward provided by the discriminator, the problem becomes a min-max optimization problem, which can lead to training instabilities and poor sample efficiency. While demonstrating state-of-the-art performance in simulated environments, this approach was demonstrated only for short-horizon small-scale tasks in real environments.

Another group of methods recovers a reward function based on the set of pre-recorded expert demonstrations. This approach is attractive for real-world robotics applications since it allows to learn the reward function offline without a need to interact with the environment. In literature, two approaches are investigated for offline reward prediction: 1) reward as a measure of discrepancy between the expert and agent behavior, and 2) reward associated with a stage of progress in task completion. In the first approach, expert demonstrations are considered to be finite distributions. The reward is based on a distance measure between the expert and agent distributions. Dadashi et al. (2021) use the Wasserstein distance as a measure between the state-action distributions of the expert and the agent. Unlike f-divergences, the Wasserstein distance Rubner et al. (2000) is based on the geometry of the metric space it operates on. To avoid excess computation the authors suggest minimizing the upper bound of Wasserstein distance. Upper bound means that greedy coupling of state-action pairs is used instead of optimal coupling.

Sermanet et al. (2017b) associate the reward with a stage of progress in task completion. This could be implemented by explicit goal discovery as by Schmeckpeper et al. (2020). Sermanet et al. (2017b) uses unsupervised clustering of image sequence based on the similarity measure between the frames, thus discovering the task stages. At deployment, image classification provides prediction about the stage of the process. The reward is assigned as a difference between the features of the test sample and the mean features of the stage cluster. The difference is then multiplied by two to the power of the stage number. This method was demonstrated on door opening and water pouring tasks in controlled laboratory conditions with almost no variation in visual conditions.

Many research works in RL for real-world applications use sparse reward indicating whether the task was accomplished successfully or not (Vecerík et al., 2019; Lee et al., 2020). We refer to it as a terminal reward. Several works train a reward function as a classifier of the final observation, predicting whether it was successful or not. While the terminal reward is sufficient for short-horizon tasks, it does not help in long-horizon large-scale tasks. Singh et al. (2019) teach the robot manipulation skills while interacting with a human and asking for a manual reward label of the observed states. Baseline reward is provided as a classification of the current state to be the final successful state. During the training, the robot queries the human to provide a label for previously-unlabeled states with the highest probability of success according to the classifier. Although the robot succeeds in learning, it is unclear how this approach would scale to the applications with a much larger state-space.

We focus on offline reward estimation from visual observations and stage-based reward, similar to Sermanet et al. (2017b). We made this choice since in our application we are dealing with the long-horizon task with a large state space.

*Visual representations–*in this work, we are interested in reward estimation from vision. Some robotics applications use pre-trained deep Convolutional Neural Network (CNN) features. For example, Sermanet et al. (2017b) uses the Inception network Szegedy et al. (2016) pre-trained for ImageNet classification Deng et al. (2009). In a number of works, generative modeling is used where a latent variable model is trained to model a latent distribution (Finn et al., 2015; Higgins et al., 2017; Lee et al., 2020). The latent variables are utilized as representations. The latent models are usually trained together with policy or other goal optimization while interacting with the environment which is impractical in real-world applications. These representations try to capture the variations related to all the underlying factors in task learning.

The representations can be trained while optimizing a contrastive loss (Schroff et al., 2015; van den Oord et al., 2018; Belghazi et al., 2018) with user-defined information. This means that a developer has to identify the factors for which the variation is modeled. For example, Sermanet et al. (2017a) proposes an approach to robotic behaviors training from unlabeled videos recorded from multiple viewpoints using time-contrastive representations. The contrastive loss tries to minimize the distance between the frames belonging to the same time window and maximize the distance to the frames outside the time window. In our previous work Yang et al. (2020), contrastive representations are used to train a vision-based imitation learning controller for autonomous pile loading. We train the representations in a Siamese neural network classifying the successful and unsuccessful pile-loading outcomes. We minimize the distance between the inner-class representations and maximize the distance between the outer-class representations. A similar approach was used in representation learning for the peg-in-hole task in Vecerík et al. (2019) ^1^.

Recently, actionable representations have been proposed to capture the variations that are important for decision making (Ghosh et al., 2019). This is implemented by comparing the actions taken by a goal-conditioned policy for two different goal states. If two goal states require different actions, then they are functionally different and vice-versa. The representations are learned such that Euclidean distance between states in representation space corresponds to actionable distances between them. The actionable distances capture the differences between the actions required to reach the different states based on Kullback–Leibler (KL) divergence. In Dwibedi et al. (2018) the actionable visual representations are learned based on the contrastive loss.

We will investigate the time-contrastive, pre-trained deep CNN features, and depth as well as Histogram of Oriented Gradients (HOG) representation, as a representative of classical edge-based features. These representations seem practical in real-world applications since they do not require training of policy together with the representations.

*Autonomous pile-loading state-of-the-art–*most of the autonomous pile loading works adopt heuristics (Fernando et al., 2018) or are model-based (Sotiropoulos and Asada 2019), and are experimented only in a simulator (Fernando et al., 2018) or toy-scale setups (D. Jud et al., 2017; Sotiropoulos and Asada 2020), which cannot capture the complicated phenomena of the real-world problem. Model-based approaches succeed in many robotics applications. However, in pile loading, the interaction between the bucket and the material is hard to model accurately. Several works attempt to learn this interaction using learning from demonstrations. Dadhich et al. (2016) fit linear regression models to the lift and tilt bucket commands recorded with a joystick. R. Fukui et al. (2015) use a neural network model that selects a pre-programmed excavation motion from a dataset of motions. Dadhich et al. (2019); Halbach et al. (2019); Yang et al. (2020) report real experiments of autonomous scooping with a real-scale Heavy Duty Machine (HDM). Dadhich et al. (2019) propose a shallow time-delay neural network controller. The controller uses the joint angles and velocities as inputs. After outdoor experiments, the authors conclude that for different conditions the network controller needs to be retrained. Halbach et al. (2019) train a shallow neural network controller (NNet) for bucket loading based on the joint angles and hydraulic drive transmission pressure. Two of our recent works by Yang et al. (2020) and Yang et al. (2021) present the state-of-the-art data-driven controller learning demonstrated on real-world pile-loader. Despite demonstrated successful performance in tested conditions and robustness against slight variations in weather conditions, the imitation learning-based, i.e., behavior cloning methods, by construction are not able to provide online adaptability to the variable conditions.

Several recent works started investigating the applicability and limitations of the reinforcement learning framework in autonomous excavation and pile-loading. Since the heavy-duty machine job mainly involves interaction with the material, RL seems a promising solution if only its limitations are addressed to make the system practically feasible. Egli and Hutter (2021) train in simulator an RL controller for the end-effector trajectory tracking of a real excavator. The training utilizes pre-recorded task demonstrations and was applied for motions generation in the air and with soil interaction in a grading task. Backman et al. (2021) present an approach to learn bucket-filling behavior for an underground loader in a simulated environment. As a reward, the authors use the bucket filling rate and energy consumption of the machine. Kurinov et al. (2020) train an agent for earthmoving in a simulated environment with sophisticated multi-body dynamics modeling. The reward function depends on the amount of simulated soil loaded and unloaded from the bucket. Azulay and Shapiro (2021) train an RL-based controller without pre-recorded demonstrations for bucket-filling first in a simulator and then test it on a toy set-up. The training in the simulator takes 3 hours and the reward depends on whether the wheel-loader followed the necessary stages of the task and the amount of loaded soil.

Current state-of-the-art works on RL for learning excavation or pile-loading are mainly in simulated or toy environments. The real-world scenarios differ from simulation by a much larger state space with high stochasticity, the longer horizon of the tasks, difficulty in defining a reward function. To make a transfer to the real-world machines, progress should be made in 1) Rl algorithms to guarantee the sample efficiency of the methods, 2) in learning of appropriate state representations from multi-modal sensors to capture the most relevant environmental conditions, and 3) proper approaches to reward estimation that would be both sample-efficient and robust to varying conditions. In the discussed works, the reward is mainly defined using the progress of the machine through the stages of the task and the amount of the material in the bucket. In the real world, one way to follow the machine’s progress is to identify the stages automatically, for example, by observing the environment. In this paper, we address the problem of reward estimation from vision by studying the available visual representations for stage-based reward estimation both for sparse terminal reward and intermediate stage reward.

## 3 Test-Study: Autonomous Pile Loading

### 3.1 Problem Statement

In this paper, we adopt the finite Markov Decision Process (MDP) as an abstraction for the problem of goal-directed episodic learning (Sutton and Barto (2018)). This is a standard abstraction in reinforcement learning literature. An autonomous agent learns through interaction with an environment. At each time step *t* = 0, 1, … *T* the agent receives the state of the environment *s*_*t*_ ∈ *S*, selects an action to perform *a*_*t*_ ∈ *A*(*s*), and receives a feedback from the environment which is called a reward $R_{t+1}\in R$ . The reward received at each time step is called an immediate reward and the goal of the agent is to maximize the cumulative reward, which is defined as the sum of rewards obtained within the time horizon *T*. A discount factor can be applied to each immediate reward which regularizes how much the agent values immediate reward versus future rewards. The cumulative reward received by the agent can be expressed as

$$r=\overset{T}{\underset{1}{\sum }}\gamma^{\left(t-1\right)}R_{t}\left(s_{t-1}\right),$$ (1)

In this work, a set of consecutive visual observations $D={\left\{o_{n},l_{n}\right\}}_{n=1}^{N}$ contains pairs of *o*_*n*_ visual observations and labels *l*_*n*_. *N* is the total number of observation-label pairs available. The visual observation is an RGB left-frame image of a stereo camera providing an ego-centric view. To avoid introducing new notation, we use *R*_*t*_ (.) to define the mapping from state or observation to reward. We thus learn an approximator function $\tilde{r}=R_{t}\left(o_{t-1}\right)$ - a predictor learnt from visual input. In long-horizon tasks, especially in real-world problems, it is beneficial to link the reward to the progress in task completion. In our framework, the visual representations are learned from the intermediate and terminal stage classifiers. The intent is to study what visual representations are useful in highly varying conditions of field robotics applications. Additionally, a recent study by Stooke et al. (2021) showed that learning of visual representations and policy separately is more beneficial in an imitation learning task.

More specifically, beside the state observations *o*_*t*_ and transition dynamics that governs environment dynamics, we assume subgoals for our long horizon task together with a final goal-we call these stages. We also assume these sub-goals or intermediate stages are sequential, that is, one needs to be performed before the other for the system to succeed. We denote them by *S*_0_, *S*_1_, *S*_2_, *S*_*T*_, where *S*_*T*_ is the terminal stage. We use supervised methods, and therefore the stages define classes which are labeled manually. The reward *r* is associated with the stage of the work process the machine is performing. Each time step the system associates the visual observation *o*_*t*_ of the machine with the stage of the process using classification model. We have two tasks: prediciting the work process stage and whether the task has completed (terminal stage). Thus, we have two training sets: for stage prediction *D* = {(*f* (*o*_*t*_), *c*_*t*_): *c*_*t*_ ∈ *C* = {0, 1, 2}} and terminal stage prediction *G* = {(*f* (*o*_*t*_), *c*_*t*_): *c*_*t*_ ∈ *C* = { − 1, 1}}. We refer to the prediction about the stage as stage-based reward $R_{t}^{S}\left(o_{t-1}\right)$ and the prediction about completion as terminal stage reward $R_{t}^{E}\left(o_{t-1}\right)\left.\right)$ . Following Eq. (1), the cumulative reward is computed as follows:

$$r=\overset{T}{\underset{1}{\sum }}\gamma^{\left(t-1\right)}\left(R_{t}^{S}\left(o_{t-1}\right)+R_{t}^{E}\left(o_{t-1}\right)\right),$$ (2)

### 3.2 Choice of Visual Representations

In this section, we describe the visual features that we have investigated for training a classification model for sub-stage and final state prediction. The first step of our classification pipeline is to compute an embedding $f\left(o_{t}\right)\in X$ , which will be passed to the classification stage. In this, X denotes the space of features. Next, we will present methods that we have investigated for learning of the embeddings for the intermediate stage reward, followed by those for the terminal stage reward. In this work, we will use the terms embedding and feature interchangeably since the embedding *f* (*o*_*t*_) acts as a feature input in our system.

#### 3.2.1 Intermediate Stage-Based Reward

In this section, we will introduce several state-of-the-art embeddings used for intermediate stage reward. We investigate the pretrained deep CNN features, time-contrastive representations, depth, and Histogram of Oriented Gradients (HOG) descriptors. We use the same class labels as described above for feature learning. These labels contain manually specified stages.

*Selected deep visual features (VGG) -* to extract deep feature embedding $f\left(o_{t}\right)\in X$ in our experiments, we used a pretrained VGG model (Simonyan and Zisserman (2015)). Following the same approach, we applied a separate selection procedure to identify the most discriminative features for the classification task. For this, we computed the mean and standard deviation for each feature *i* for each class on the training set

$$z_{i}=\alpha |\mu_{i}^{+}-\mu_{i}^{-}|-\left(\sigma_{i}^{+}+\sigma_{i}^{-}\right),$$ (3)

where $\mu_{i}^{+}$ and $\sigma_{i}^{+}$ are the mean and standard deviation of the features within the same class and $\mu_{i}^{-}$ and $\sigma_{i}^{-}$ those of the rest of the classes. The labels of the classes are known from the ground truth data. We selected 200 features with a top *z*_*i*_ score in each class. This led to 29 deep features with top scores in all classes. The value 200 was selected empirically such that there are enough features with high score in all classes. One drawback of this approach is that the selection procedure might suffer from the drift of feature space when the environment changes.

*Time-constrastive representations (TCR) -* TCR has shown to provide robust embedding maps for imitation learning tasks, as well as object, face, action recognition and alignment Sermanet et al. (2017a); Schroff et al. (2015). Here we describe our experiments with TCR. To capture the dynamic nature of the task, an input to the classification system at time *t* is *x*_*t*_, a stack of *d* image frames

$$x_{t}=\left(o_{t-d+1},\ldots ,o_{t-1},o_{t}\right),$$ (4)

where *o*_*t*_ is an rgb image observations. In our experiments, the size of a single observation image *o*_*t*_ was reduced to 64 × 64; the number of frames *d* was 5. Each step, we choose a random input sequence *I*_*t*_ = (*x*_*t*_, *x*_*t*+1_, … , *x*_*t*+*n*_) in the training set and sample a random anchor input stack *x*_*i*_: *t* ≤ *i* ≤ *t* + *n*. The positive input stack $x_{i}^{+}$ is sampled from a window (*x*_*i*−*L*_.*x*_*i*+*L*_), where *L* is a fixed window size. The negative sample $x_{i}^{-}$ is randomly chosen outside this window within sequence *I*_*t*_. The anchor, the positive, and the negative inputs are added to the training set.

Let *f*_*θ*_(*x*_*i*_) denote the feature extraction (embedding) network with parameters *θ*. With an anchor *x*_*i*_, a positive sample $x_{i}^{+}$ , and a negative sample $x_{i}^{-}$ , we formulate the loss to draw positive samples closer to the anchor in the feature space and negative samples further away. Thus, the embedding *f* (*x*_*i*_) shall satisfy Sermanet et al. (2017a):

$$∥f\left(x_{i}\right)-f\left(x_{i}^{+}\right){∥}_{2}^{2}+\alpha <∥f\left(x_{i}\right)-f\left(x_{i}^{-}\right){∥}_{2}^{2},\forall \left(f\left(x_{i}\right),f\left(x_{i}^{+}\right),f\left(x_{i}^{-}\right)\right)\in \Gamma ,$$ (5)

where *α* is an empirical parameter and Γ is the set of all possible triplets in the training set. We used a state-of-the-art video frame interpolation method DAIN (Depth-Aware Video Frame Interpolation) Bao et al. (2019) to robustify the training since our training set is limited.

*Selected HOG features -* We also experimented with HOG, as a representative of classical methods. HOG demonstrated good performance in visual recognition tasks Lowe (2004); Dalal and Triggs (2005). HOG stands for the Histogram of Oriented Gradient descriptors. The HOG descriptor (embedding) characterizes an image by the distribution of local intensity gradients or edge directions. This information was shown to be rather sufficient even without precise knowledge of the corresponding gradient or edge positions. The embedding *f* (*o*_*t*_) is computed by dividing an image into small spatial regions, for each region accumulating a local histogram of gradient directions or edge orientations over the pixels of the region. The combined histogram entries form the embedding. To improve the invariance to illumination, and other conditions, the local responses within a fixed block are contrast-normalized using L1-regularization. Let *v* be the unnormalized descriptor vector, norm *v*_1_ be its 1-norm *η* be a small constant. Normalization is done by dividing each value in the vector by its norm: $v_{n}=\frac{v}{\left(\mathrm{norm}v_{1}+\eta \right)}$ We use *sklearn*1 implementation to compute the HOG representations.

*Depth features -* to obtain the depth image we used MonoDepth, the state-of-the-art depth computation pretrained on the KITTI dataset for outdoor environments2. We then define the features to be an array of depth values in fixed image locations.

#### 3.2.2 Sparse Terminal Reward

We define a sparse reward signal that indicates whether the task was completed in the current episode. Practically, at each time step, it predicts whether the terminal stage has been reached. The approach to predict the terminal stage is similar to the TCR. For this, we use task-specific visual features that we previously trained and demonstrated in behavior cloning of pile loading task Yang et al. (2020). There we trained the features on summer data and were testing during the summer/autumn period. The training objective was that the network should learn to distinguish the successful samples (the bucket is full and task accomplished) from unsuccessful samples (the bucket is empty or task is unfinished). For the main target, the standard cross-entropy loss worked well. However, in addition to the cross-entropy loss for classification, a contrastive loss Hadsell et al. (2006) was added to constrain visual feature extraction. The features within the positive examples should be close to each other in the feature space and far from the negative examples. The samples were labeled manually as described in the experimental section. The contrastive loss has been used in one-shot learning tasks and metric learning tasks where feature distances become important Koch et al. (2015) ^2^.

Here, the learning process is similar to the time-contrastive representations (TCR). The difference is that in TCR we sample a triplet of instances and here we sample a pair. Let *x*_1_, *x*_2_ ∈ *Z* be the input samples. As in TCR, we use a stack of RGB images as input. In TCR we used only the time-contrastive loss since the sampling of data was based on the time window approach and not based on identified classes. Here, positive samples belong to the terminal stage and the negative ones do not. We train the embeddings in the Siamese classification network *f*_*θ*_(*x*) where the standard cross-entropy loss *L*_*ce*_ (Bishop (2006)) is combined with the contrastive loss *L*_*c*_ in the following way:

$$L=\lambda_{1}L_{ce}\left(f_{\theta }\left(x\right)\right)+\lambda_{2}L_{c},$$ (6)

where *λ*_1_ = 0.6 and *λ*_2_ = 0.4 denote the weight of the losses.

The contrastive loss is defined by

$$L_{c}=\left\{\begin{array}{cc} {\max\left(0,m-D\left(x_{1},x_{2}\right)\right)}^{2},\; & \text{if}\;\;x_{1}\in Z^{-},x_{2}\in Z^{+}\\ D{\left(x_{1},x_{2}\right)}^{2},\; & \text{if}\;\;x_{1},x_{2}\in Z^{+} \end{array}\right.$$ (7)

$$D=∥f_{\theta }\left(x_{1}\right)-f_{\theta }\left(x_{2}\right){∥}_{2}$$ (8)

where *x*_1_ and *x*_2_ are input into a shared-weights siamese network and *D* (*x*_1_, *x*_2_) is the Euclidean distance between the features. By minimizing the above loss function, the network parameters are trained in a way that features of inputs within the positive samples come closer and those of the inputs belonging to different sets get farther. Parameter *m* (*m* > 0) is a margin value based on the spring model analogy (Hadsell et al. (2006)). The margin defines a radius within which the negative samples contribute to the loss function. The value of m depends on the distribution of features in the contrastive classes. We selected empirically *m* = 0.3. *m* corresponds to *α* parameter in TCR. We used also the video frame interpolation DAIN Bao et al. (2019) and image data augmentation to generate more training examples and robustify the training.

### 3.3 Choice of Classification Methods

In this section, we describe the classification methods that we have used to learn the classification model.

*K-Nearest Neighbor (KNN) -* KNN is a straightforward non-parametric method for classification where no assumption is made on the distribution of the data (Duda et al. (2012)). The decision about each new test sample is made based explicitly on the training samples. Its simplicity is an advantage of the method, whereas computational complexity at the test time is the downside. To classify a new test observation *f* (*o*_*i*_), we use Euclidean distance to identify a set of *M* nearest neighbors in the feature space. We assign the class label of the majority of the neighbors to the test sample.

*Support Vector Machine (SVM) -* we use the multi-class SVM formulated as one-versus-one classification. The basic SVM is a two-class classification approach which we explain further. We will therefore formulate it first for the two-class problem of terminal reward classification. The SVM uses a linear discriminant function of the form

$$y\left(f\left(o_{i}\right)\right)=w^{T}\phi \left(f\left(o_{i}\right)\right)+b,$$

where *ϕ*(*f* (*o*_*i*_)) denotes a fixed feature-space transformation of our embedding (e.g. RBF or polynomial) and *b* is a bias term. The new sample *f* (*o*_*i*_) is classified according to the sign of *y* (*f* (*o*_*i*_)). Assumption is that there exists at least one choice of parameters *w* and *b* such that two classes are separable in high-dimensional space of *ϕ*(*f* (*o*_*i*_)): *y* (*f* (*o*_*i*_)) > 0 for samples having *c*_*i*_ = 1 and *y* (*f* (*o*_*i*_)) < 0 for samples having *c*_*i*_ = −1.

To train *w* and *b*, the following objective function is used

$$\underset{w,b}{\mathrm{argmax}}\left\{\frac{1}{\left\|w\right\|}\underset{n}{\min}\left[c_{n}\left(w^{T}\phi \left(f\left(o_{n}\right)\right)+b\right)\right]\right\}$$

It maximizes the margin between two classes, where the margin is defined by the supporting hyperplanes separating the classes.

This can be transformed into the quadratic programming problem and solved, for example, with the method of Lagrangian multipliers (please, see Bishop (2006) for further details). We used the sklearn3 multi-class implementation.

*Random Forest (RF) -* RF classification is more robust to the data intrinsic ambiguities when different output values might be associated with the same input values. (Criminisi et al. (2012)). Each tree learns only from a partial set of features and therefore can learn important cues about the different stages. The random forest ${F_{r}}_{f}$ is a collection of decision trees:

$${F_{r}}_{f}=\left\{T_{\theta_{m}}^{m};,\;m=1,2,\ldots ,M\right\}$$

*θ*_*m*_ denotes the parameters of each tree $T^{m}$ . A decision tree is a special graph structure consisting of a set of questions hierarchically organized. By answering the question a decision tree can evaluate a property of a sample or identify its class or category. Parameters of these questions or rules are the parameters of a decision tree. Each decision tree in the forest classifies a sample according to its own rules and based on a sub-set of training data. During the training, each tree is trained separately. In our work, given the input feature *f* (*o*_*i*_), the classification result produced by the random forest ${F_{r}}_{f}$ is:

$${F_{r}}_{f}\left(x_{t}\right)=\frac{1}{m}\overset{M}{\underset{j=1}{\sum }}T_{\theta_{j}}^{j}\left(f\left(o_{i}\right)\right)$$ (9)

that is, the output of the random forest is the average of all class probabilistic predictions produced by the trees.

The training of ${F_{r}}_{f}$ is performed as following: 1) draw a bootstrap data *D*_*bs*_ from the training set; 2) grow a classification tree $T^{m}$ to the bootstrapped data *D*_*bs*_, fit each tree until the maximum depth is reached. Each tree $T^{m}$ is trained as following: 1) randomly select *n* features from the *k* features (*n* < *k*); 2) pick the best variable split-point among the *n*; 3) split the node into two child nodes. Each node of each tree $T^{m}$ in our implementation is trained by minimizing the Gini impurity. For any node *j* and class *C*_*i*_, *p*_*j*_ (*C*_*i*_) is a fraction of samples at *j* that belongs to class *C*_*i*_. With a total number of classes *M*, the Gini impurity in node *j* is the probability of incorrectly classifying a randomly chosen element in the dataset:

$$\overset{N}{\underset{i=1}{\sum }}p_{j}\left(C_{i}\right)\left(1-p_{j}\left(C_{i}\right)\right).$$

## 4 Experiments

The goal of the performed experiments was to explore the performance of the currently used representations for stage-based reward evaluation in highly varying weather conditions. We demonstrate the results for the outdoor pile loading task. At the beginning of this section, we introduce the set-up details and task description, including data collection and resulting datasets. After that we 1) present the results of stage discovery using the studied visual representations and classification methods; 2) test the task-specific visual features for terminal stage prediction, and 3) demonstrate how cumulative reward is computed using the stage information and perform the qualitative analysis of the results.

### 4.1 Set-Up

*Wheel-loader -* The autonomous scooping was implemented on a robotic wheel-loader, a so-called GIM machine. It has the mechanics of a commercial wheel loader (Avant 635) and power transmission and controllers are custom-made at Tampere University. The bucket is positioned in the vertical plane by two joints, the boom joint and the bucket joint, and in the horizontal plane by drive (throttle/gas) and articulated by a frame steering mechanism4. The GIM machine is equipped with various sensors including, for example, GNSS (Global Navigation Satellite System), wheel odometry, IMU (Inertial Measurement Unit), and pressure sensors.

**FIGURE 4:**
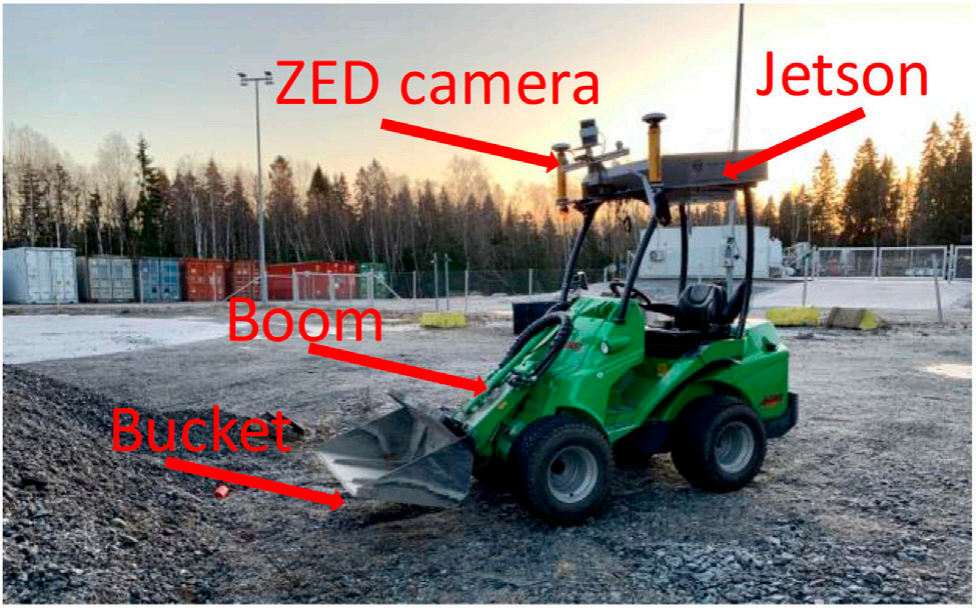
Robotic set-up at test site.

*Vision -* We used a ZED stereo camera to get visual feedback. The ZED camera images of 2560, ×, 720 resolution were captured at the frame rate of 15*fps*. The left RGB image was used to generate the embeddings that are used as features in stage classification. The experiments were performed at the outdoor test site (see Figure 4).

*The control system -* is composed of multiple layers. In the lowest level (digital and analog I/O and CAN), industrial micro-controllers implement power management and basic safety functions. In the PC control level, a target PC runs Simulink Real-time models, which run real-time tasks such as localization. Sub-systems communicate low-level sensor data and control commands via UDP protocol running on a Jetson AGX Xavier (8-Core ARM v8.2 64-bit NVIDIA Carmel CPU and 512-core NVIDIA Volta GPU with 64 Tensor Cores) on-board. All the data collection, learning, and closed-loop control are implemented on the Jetson PC.

*Workflow of experiments -* during training, we learn the models to compute the TRC representations (Sec. 3.2.1), the sparse terminal reward representations (Sec. 3.2.2), and classification models (Sec. 3.3). At test time, we follow the routine in Algorithm 1 which is performed on each sample of the test data.

Algorithm 1 Visual rewards for sequential tasks: workflow of experiments at test time.

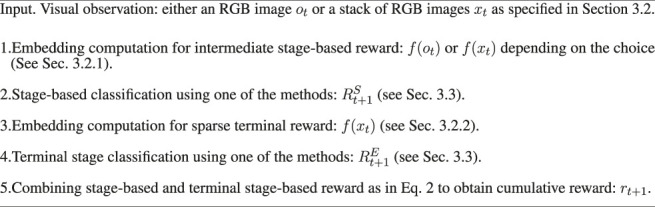

*Performance metric -* as a performance measure, we use classification accuracy in percent:

$$\text{Accuracy}=\frac{\text{Number of correct predictions}}{\text{Total number of predictions}}*100\%$$ (10)

In our work, the reward is assigned based on the classification prediction. If the predictions match the ground-truth label of the sample, they are correct.

### 4.2 Data

*The bucket filling task -* Figure 2 demonstrates three stages of the task: driving up to the pile, filling the bucket, and lifting the boom. This process takes about 30–60 s for a human operator depending on the conditions of the ground. We treat this task as an episodic, long-horizon, sequential task. Since it is a real-world task, there might be variations in operator performance resulting in noisy and non-consistent demonstrations. For example, while loading the pile (stage 2), the operator might still use gas in order to load a larger amount of load.

*Reset to initial conditions -* in all our experiments, every episode starts from the same initial conditions. The bucket is unloaded; the wheel-loader is driven a certain distance away from the pile (1–5 m); the boom is placed in a lower position, and the bucket is leveled with the ground. The process of bringing the machine to its initial state takes several minutes. In all our experiments, we automated this procedure by pre-programming the machine to reach certain joint values using a state machine algorithm. The distance the machine drives away from the pile is defined manually.

**FIGURE 5:**
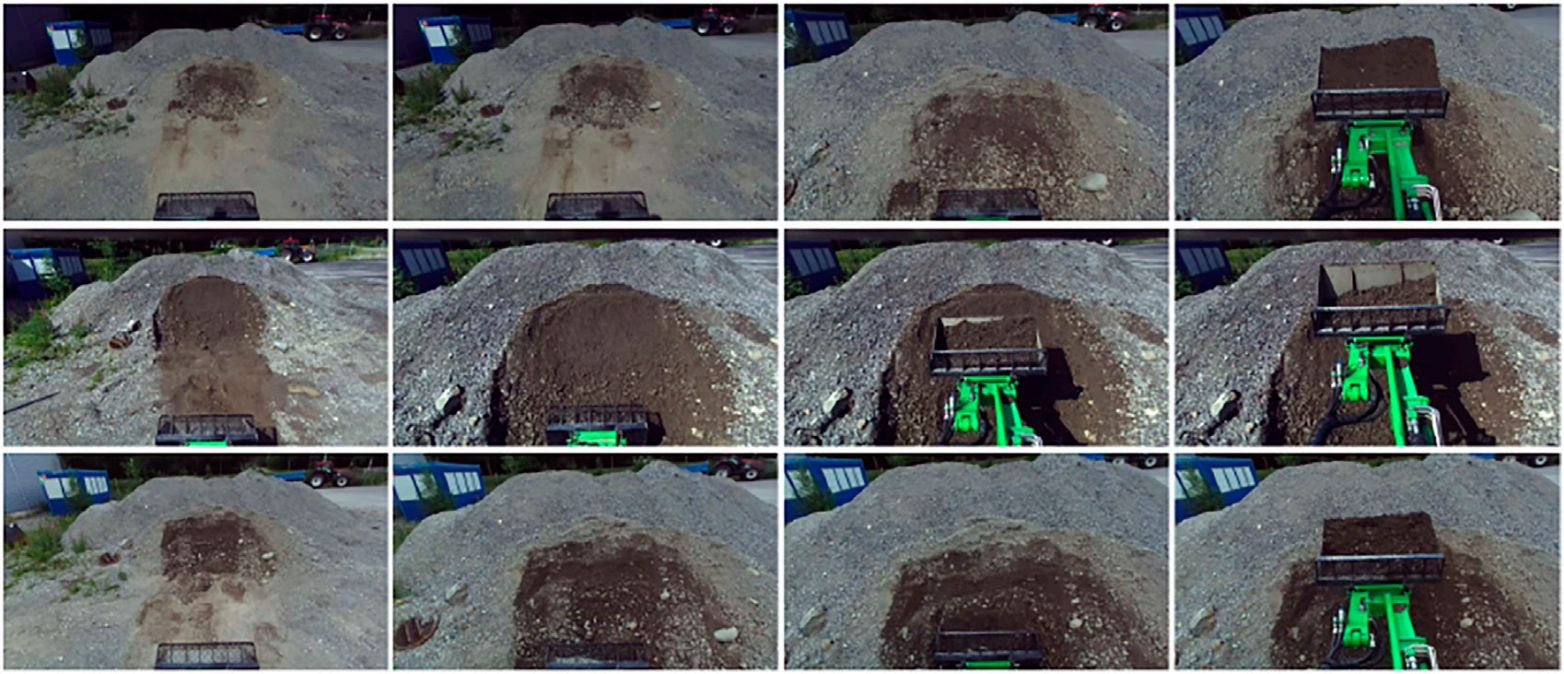
Examples of images from *D*_*summer*_.

**FIGURE 6:**
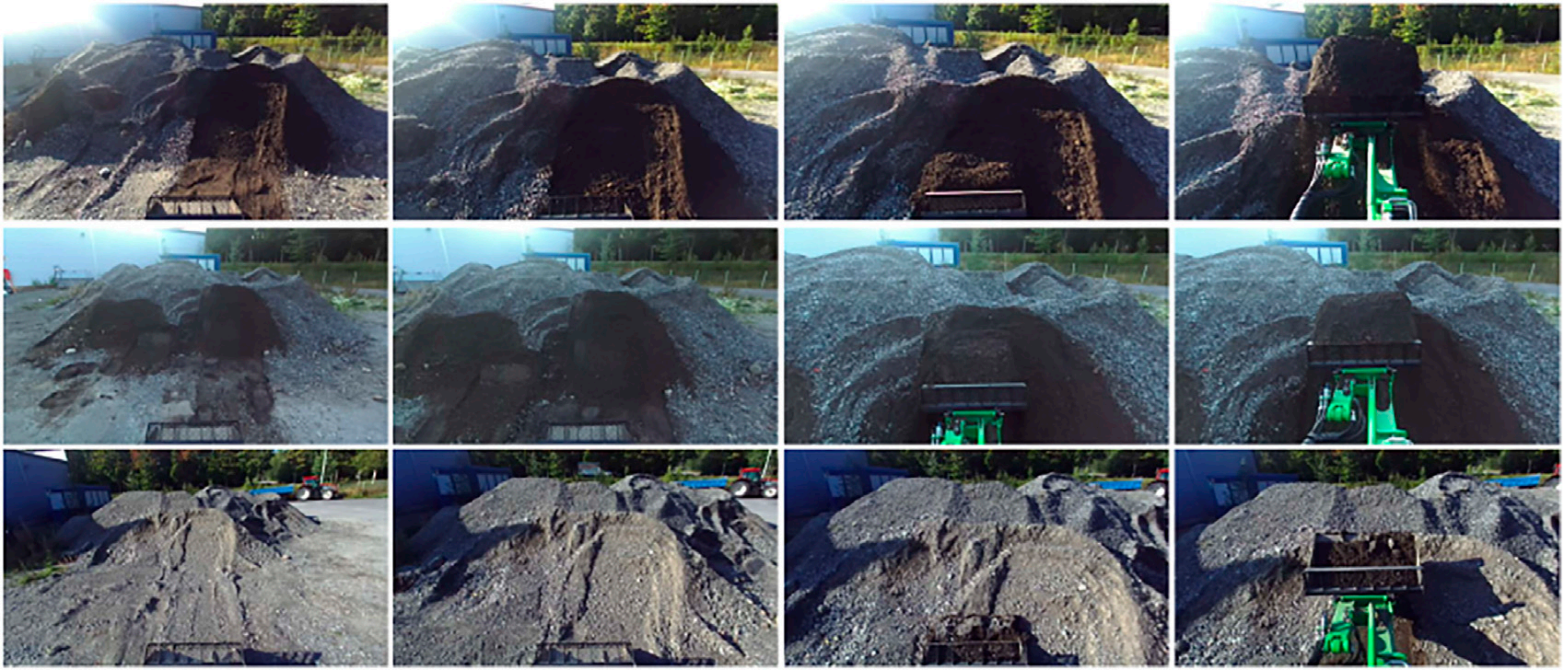
Examples of images from *D*_*autumn*_.

**FIGURE 7:**
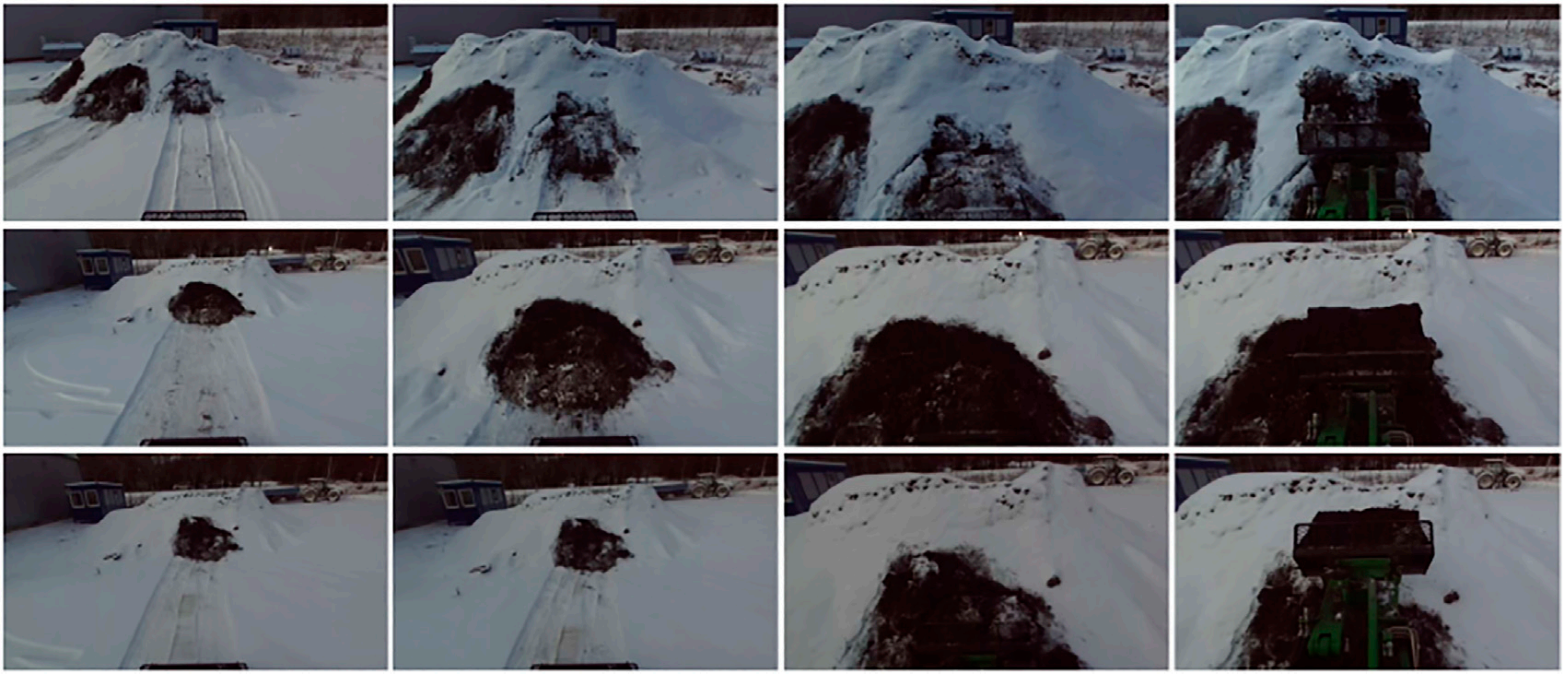
Examples of images from *D*_*winter*_.

*Datasets -* we created three sets of data:

• *D*_*summer*_: 70 human demonstrations collected during two summer days (See examples in Figure 5);
• *D*_*autumn*_: 25 roll-outs of an RF controller trained in Yang et al. (2020) (See examples in Figure 6);
• *D*_*winter*_: 25 human demonstrations collected during one snowy winter day (See examples in Figure 7).

All the samples in the dataset are successfully performed bucket filling demonstrations. The amount of load in different demonstrations might vary. The collected data includes images from the ZED camera, joint positions, pressure signals, and commands. We used the commands produced by the operator or controller to manually annotate the stages of the task and whether the terminal condition was reached. In the stage identification algorithms as well as representation learning only visual data was used. Each image was labeled by 1) one of three classes corresponding to the stage of the process, and 2) by a binary label belonging to the terminal state or not (-1 or 1)^3^ ^,^ ^4^.

### 4.3 Preliminary Experiments With Manually Assigned Rewards

In prior work, we attempted the approach by Singh et al. (2019) where the robot can query a human about the reward for a given visual observation. A baseline reward was given by a classification prediction of whether the observed state is a final successful state. In the original paper, the reward was queried for states which were predicted to be close to successful. In our implementation, the user assigned a reward manually when the robot was proceeding through the correct stages. Our task had a much longer horizon and reward classification did not provide a sufficient accuracy rate due to higher variations in visual observation. We used DDPG RL algorithm (See Lillicrap et al., 2015). In our experiment, the robot failed to converge to any solution within several hours. This experiment motivated us to study the topic of reward discovery separately.

### 4.4 Stage-Based Intermediate Reward

This section presents quantitative results of stage discovery for the pile loading tasks. The TCR representations were trained on the *D*_*summer*_. The HOG and VGG features were selected based on the *D*_*summer*_. We experiment in the following scenarios: 1) classification methods trained on *D*_*summer*_ and tested on the rest of the seasons, and 2) classification methods trained on the mixture of seasons. Scenario 1 has practical importance. In real-world industrial applications, it is desirable to collect the data once, develop models based on it, and re-use them. Scenario one tests this opportunity for visual rewards. We used 2-fold cross-validation repeated five times in our reporting of testing and training results, i.e., 50% of data was selected for training and 50% for testing which was repeated five times.

*Scenario 1 -* in Table 1 the results of stage discovery is shown when we merged the labels for stage 2 and 3 into one class. The results for using three classes were quite unsatisfactory. By reducing the number of stages (basically discovering whether the machine is driving to the pile or performing the scooping action), a more reasonable performance was achieved. The results suggest that depth and selected deep VGG features provide the most reliable cues. The results show that the time-contrasting features overfit training data and performance degrades on test data.

**TABLE 1:** Stage discovery accuracy **trained on** ***D***_***summer***_. Two stages. We used repeated 2-fold cross-validation, i.e., 50% of data was selected for training and 50% for testing which was repeated five times. Highest accuracy for each season marked in bold.

Stage discovery accuracy [%]				
		Tested on		
Feature	Classifier	*D*_*summer*_	*D*_*autumn*_	*D*_*winter*_
HOG	KNN	78	50	58
	SVM	80	55	55
	RF	79	53	59
VGG	KNN	88	**68**	68
	SVM	92	62	55
	RF	90	63	63
TCR	KNN	**99**	59	43
	SVM	**99**	67	56
	RF	**99**	67	56
Depth	KNN	84	61	**73**
	SVM	82	66	67
	RF	89	60	60

*Scenario 2 -* Table 2 contains the results when the training and testing was done on a mix set of all seasons. We compare the discovery rate for two- and three-stage labeling. As expected, the performance improved significantly when the representatives of all seasons were present in the dataset. Another reason for the improvement is that the summer data share is highest in the whole set. However, both depth and selected VGG produced the highest accuracy of more than 95%. HOG and time-contrastive representations demonstrate slightly worse performance. However, the accuracy does not drop significantly between the training and test sets.

**TABLE 2:** Stage discovery accuracy **trained on all seasons**. We used repeated 2-fold cross-validation, i.e., 50% of data was selected for training and 50% for testing which was repeated five times. Highest accuracy for each scenario marked in bold.

*Stage discovery accuracy [%]*			
Feature	Classifier	Two stages	Three starges
HOG	KNN	88	85
	SVM	74	64
	RF	82	75
VGG	KNN	95	93
	SVM	96	94
	RF	94	91
TCR	KNN	85	74
	SVM	85	77
	RF	77	69
Depth	KNN	**98**	**96**
	SVM	90	68
	RF	97	**96**

As for the classification methods, there was no significant difference between the approaches. In most cases, the KNN and RF classifier demonstrated better performance. Time-contrastive features overfitted the training data in Scenario one and demonstrated worse performance in Scenario 2. The reason for this might be that, compared to prior work Sermanet et al. (2017a), the view is ego-centric and the structure of the motion is not observed. A further study should address combining ego-centric and third-person views complementing each other.

### 4.5 Terminal Reward Estimation

In this experiment, we attempted to use the task-specific visual features previously learned and successfully demonstrated in our behavior cloning experiments (see Yang et al. (2020)). Each image received a label minus one or one, where one corresponds to task completion and minus one to the rest. Similarly to the previous section, we test two scenarios 1) trained only on summer data *D*_*summer*_ and tested on two other datasets *D*_*autumn*_ and *D*_*winter*_ and 2) trained and tested on a mixed set containing representatives of all weather conditions. A 2-fold cross-validation repeated five times was used. Table 3 contains the results of this experiment. In Scenario one although the performance drops while transferring between seasons, the average best accuracy for the test seasons was about 83%. Training and testing on the mixed set of data (Scenario 2) allowed to improve the results significantly with top accuracy of 95%. When trained and tested on the same season (summer) the learned feature demonstrates 99% accuracy for the RF classifier.

**TABLE 3:** Terminal state classification. We used repeated 2-fold cross-validation, i.e., 50% of data was selected for training and 50% for testing which was repeated five times. Highest accuracy for each scenario marked in bold.

*Step-wise accuracy [%]*										
	Trained on summer, tested on			Trained on autumn, tested on			Trained on winter, tested on			Trained and tested on mix
Classifier	*D*_*summer*_	*D*_*autumn*_	*D*_*winter*_	*D*_*summer*_	*D*_*autumn*_	*D*_*winter*_	*D*_*summer*_	*D*_*autumn*_	*D*_*winter*_	
KNN	98	76	80	78	97	92	74	65	98	95
SVM	85	66	78	82	89	94	81	82	94	84
RF	99	75	90	79	95	92	79	78	97	94

### 4.6 Combining the Stage-Based Rewards

**FIGURE 8:**
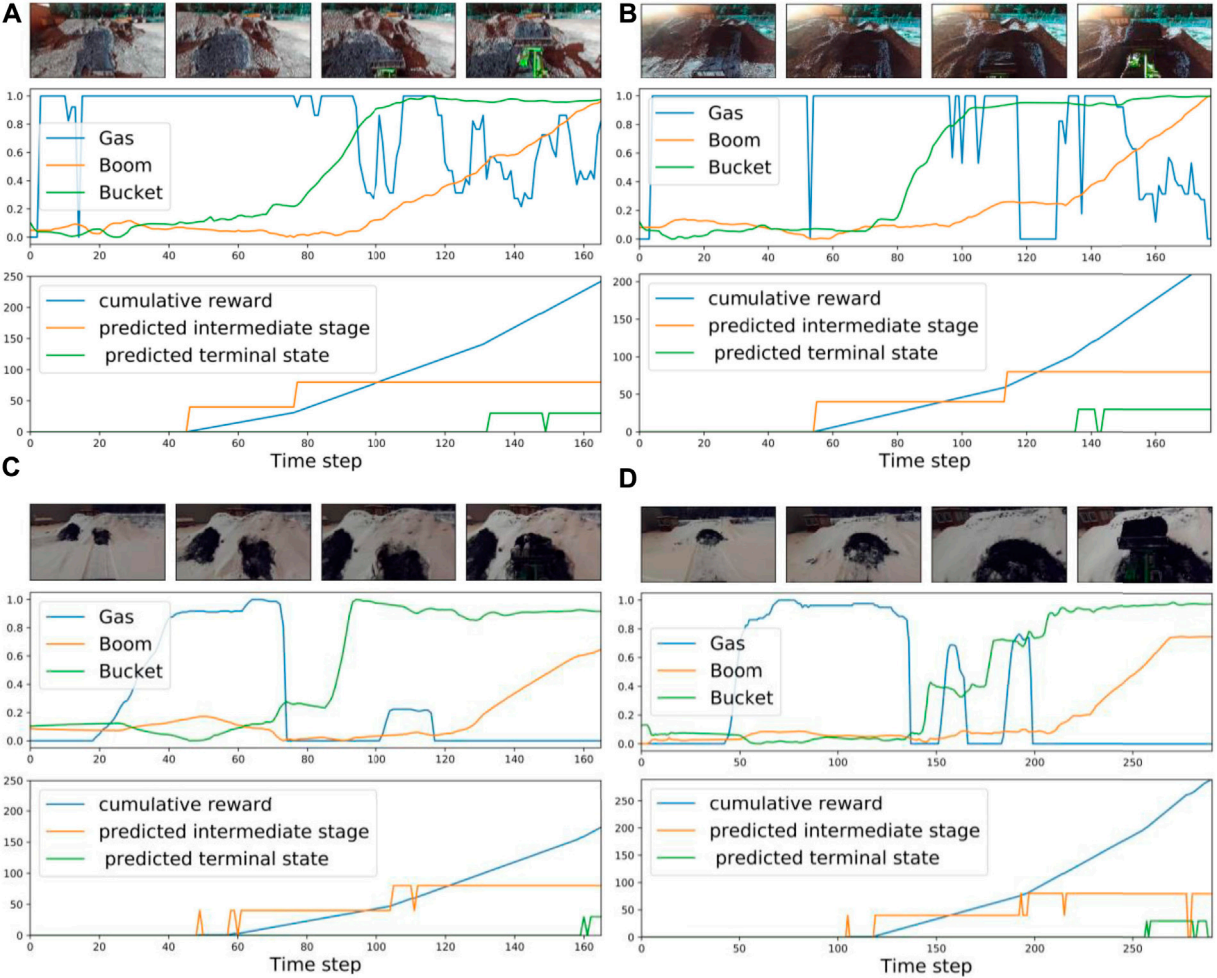
Examples of proposed cumulative reward based on the discovered stages: **(A,B)** summer, different light conditions, **(C,D)** winter, different locations.

For each visual observation *o*_*i*_, we obtain a label *c*_*i*_ of the current stage the machine is performing and a prediction $c_{i}^{T}$ whether the task has been completed. At each time step, we can sum these predictions and compute cumulative rewards as a weighted sum (see Eq. (2)). Figure 8 presents examples of cumulative rewards for summer and winter scenarios. Despite the noisy stage prediction, the cumulative reward is not much affected. The analysis of stage classification is presented in the next section.

### 4.7 Qualitative Results and Discussion

In this section, we investigate the stage prediction in detail. Every subplot in Figures 11–13 contains the stage prediction for the specified combination of a visual representation and a classification method. Each subplot presents the results for each episode in the datasets. The end of each episode corresponds to the right-most end of the *x*-axis since the duration of episodes varies.

#### 4.7.1 Stage-Based Reward

In the following, we discuss the results according to the scenarios introduced in Section 4.4.

**FIGURE 9:**
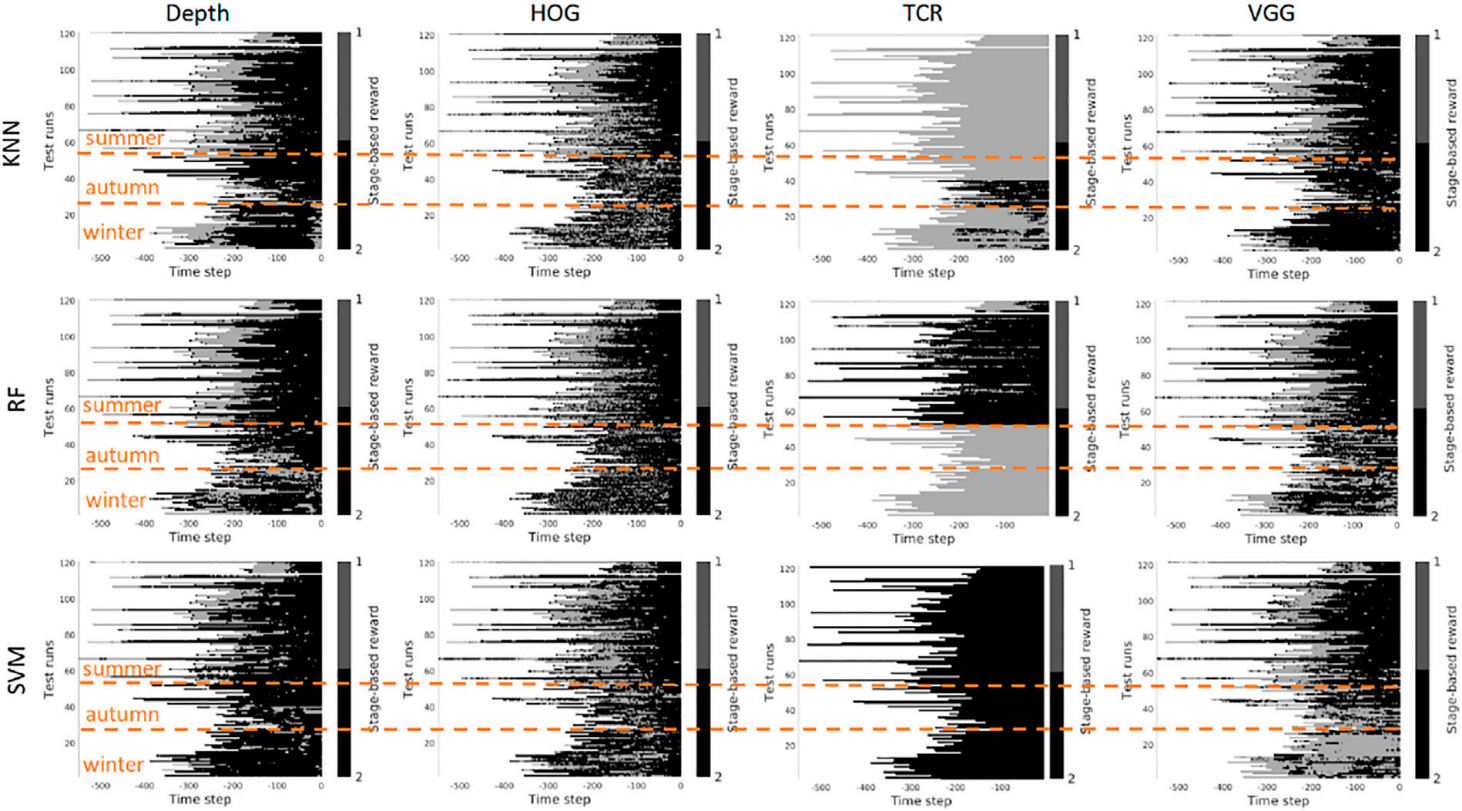
Stage-based reward plotted for all seasons. Scenario 1: trained on the summer set of data *D*_*summer*_, 2 stages. The stages proceed as follows: *S*1 − > *S*2. *x*-axis stands for the time step in reversed order to match the samples of varying length. Color bar encodes the stages: *S*1 -gray, *S*2 - black.

**FIGURE 10:**
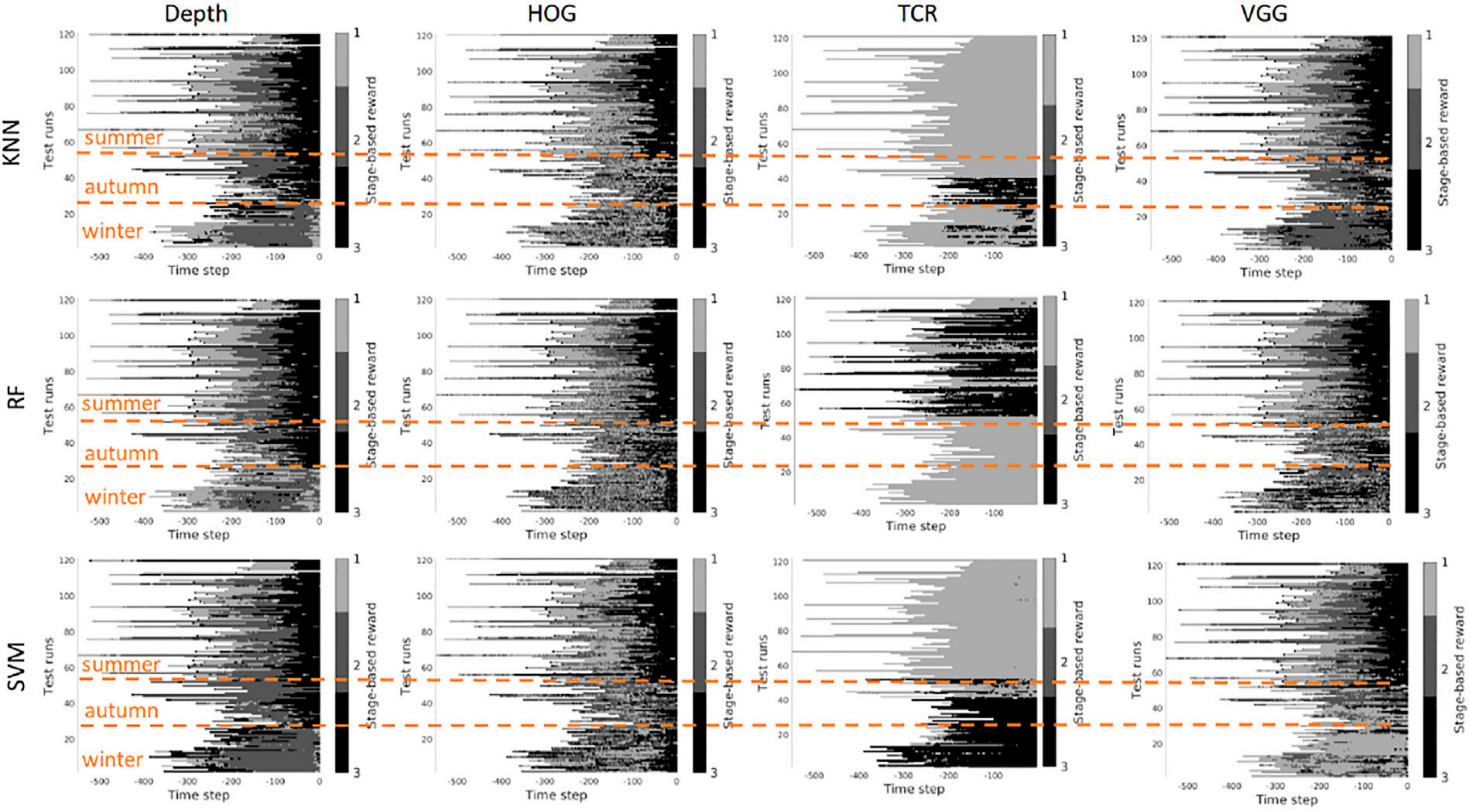
Stage-based reward plotted for all seasons. Scenario 1: trained on the summer set of data *D*_*summer*_, 3 stages. The stages proceed as follows: *S*1 − > *S*2 − > *S*3. *x*-axis stands for the time step in reversed order to match the samples of varying length. Color bar encodes the stages: *S*1 - light gray, *S*2 - gray, *S*3 - black.

*Scenario 1,* “*training on the summer data*” *-* Figures 9, 10 contain the stage prediction results for the two- and the three-stage classification task. Qualitatively and quantitatively depth features combined with KNN provide the highest accuracy compared to other combinations. Three-stage classification results in too noisy outputs when training on summer only. HOG representations, which reflect the geometrical information in the images, visually perform better than TCR and VGG representations. TCR representations do not cope with the task in our settings. This might be because we train them from scratch on a small set of data. Although we use the data augmentation technique to increase sample variation, it seems not to help in the explored task. As for the classification method, RF and KNN provide similar results, however, RF is more computationally efficient than KNN.

**FIGURE 11:**
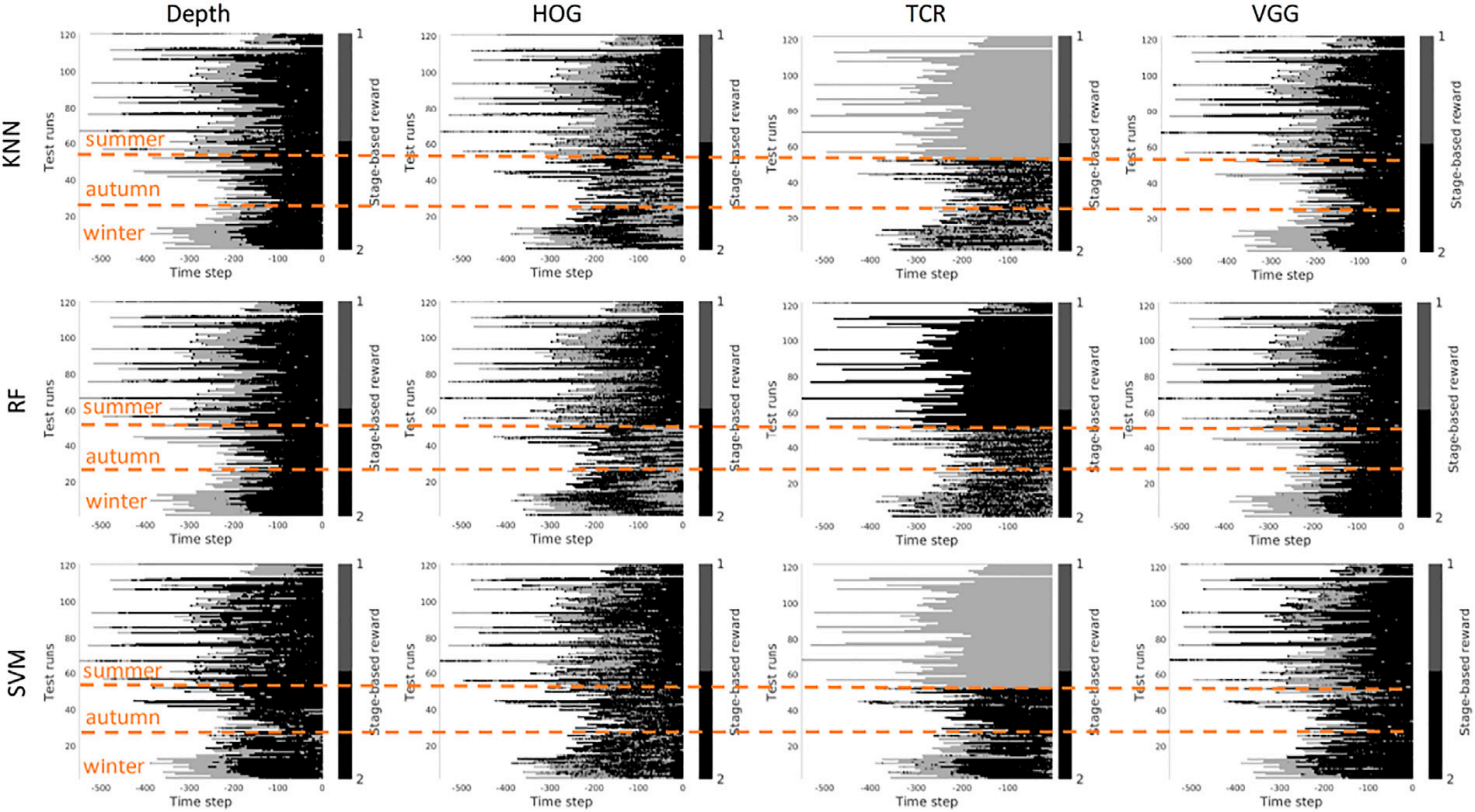
Stage-based reward plotted for all seasons. Scenario 2: trained on the mixed set of data, 2 stages. The stages proceed as follows: *S*1 − > *S*2. *x*-axis stands for the time step in reversed order to match the samples of varying length. Color bar encodes the stages: *S*1 -gray, *S*2 - black.

**FIGURE 12:**
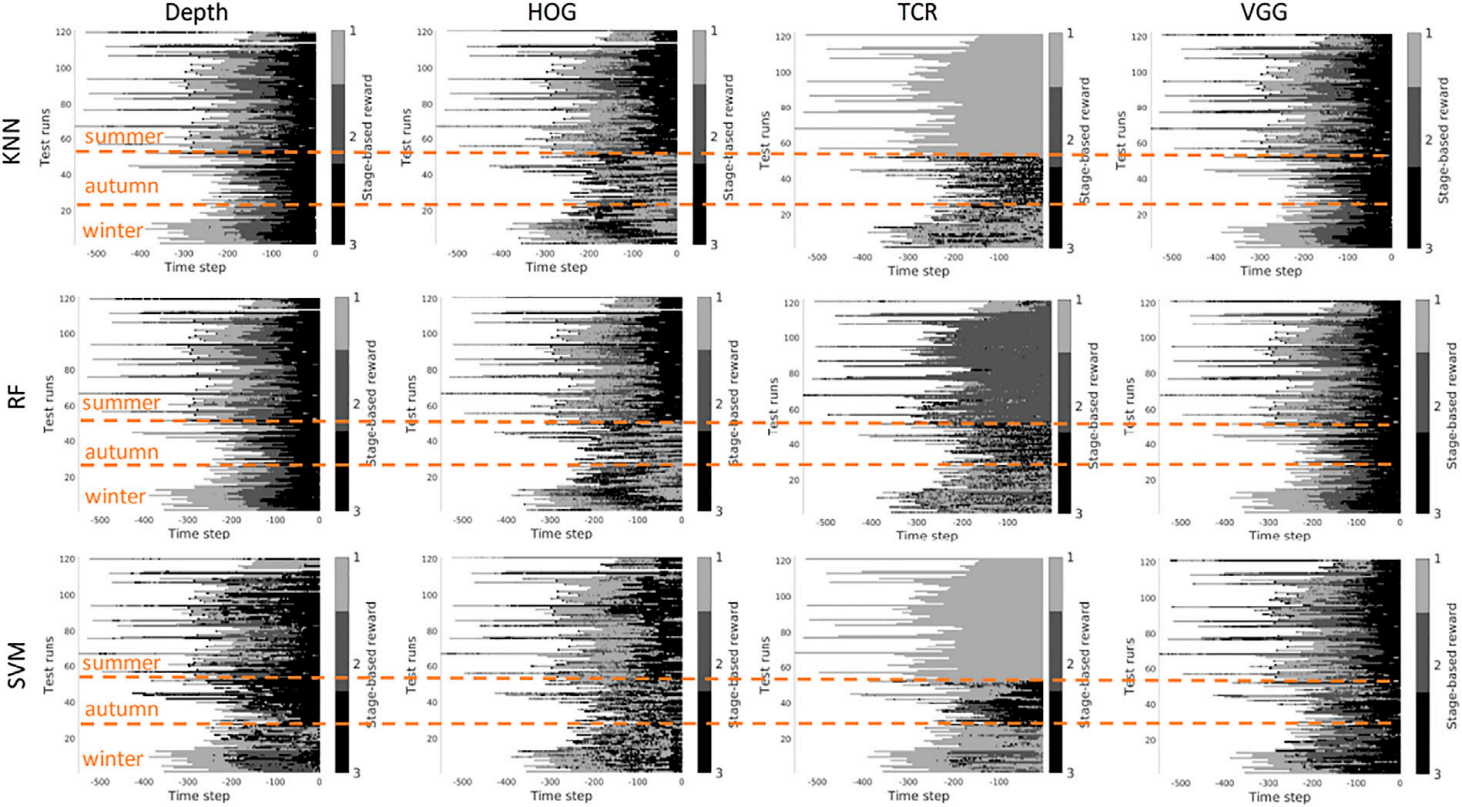
Stage-based reward plotted for all seasons. Scenario 2: trained on the mixed set of data, 3 stages. The stages proceed as follows: *S*1 − > *S*2 − > *S*3. *x*-axis stands for the time step in reversed order to match the samples of varying length. Color bar encodes the stages: *S*1 - light gray, *S*2 - gray, *S*3 - black.

*Scenario 2,* “*training on the mixed data*” *-* when training is performed on the mixed set of data (see Figures 11, 12), the results look significantly better, as expected. The depth and VGG combined with KNN demonstrate the best performance. HOG seems still better than TCR.

*Summary -* in our task settings we have only a limited set of data available. It seems that in such conditions training TCR even with augmentation does not seem to be feasible. Another consideration is that previous works demonstrated successful use of time-contrastive representations in the tasks where the whole structure of motion was visible in the collected images. And the representations were learned on the multi-view set of data. The applicability of such representations to more general settings had to be investigated. In our work, we attempted also to use HOG features. This representation demonstrated visually logical outputs (not just random performance but noisy sequential output). However, HOG features are rather heavy to compute at the run time. Depth and VGG demonstrated the best results which means that in robotics applications it is still better to rely on physical measurements. The pretrained deep VGG features seem to be able to capture the distance phenomenon and thus be closer to the depth performance.

However, three aspects have to be taken into account. 1) The ground truth labeling by a human might affect the classification results because in reality there is no clear border between the stages, and the expert that was marking the data had to make a decision where to draw a line between the stages. Therefore it makes sense to look at the qualitative results. 2) The results that we present do not contain any output filtering or smoothening which could have been implemented to avoid additional noise in the output. The noisy output can be also eliminated with a higher level logic based on, for example, the order of the stages. 3) the reward learned on the offline set of data shall be improved in learning online.

#### 4.7.2 Terminal Reward

**FIGURE 13:**
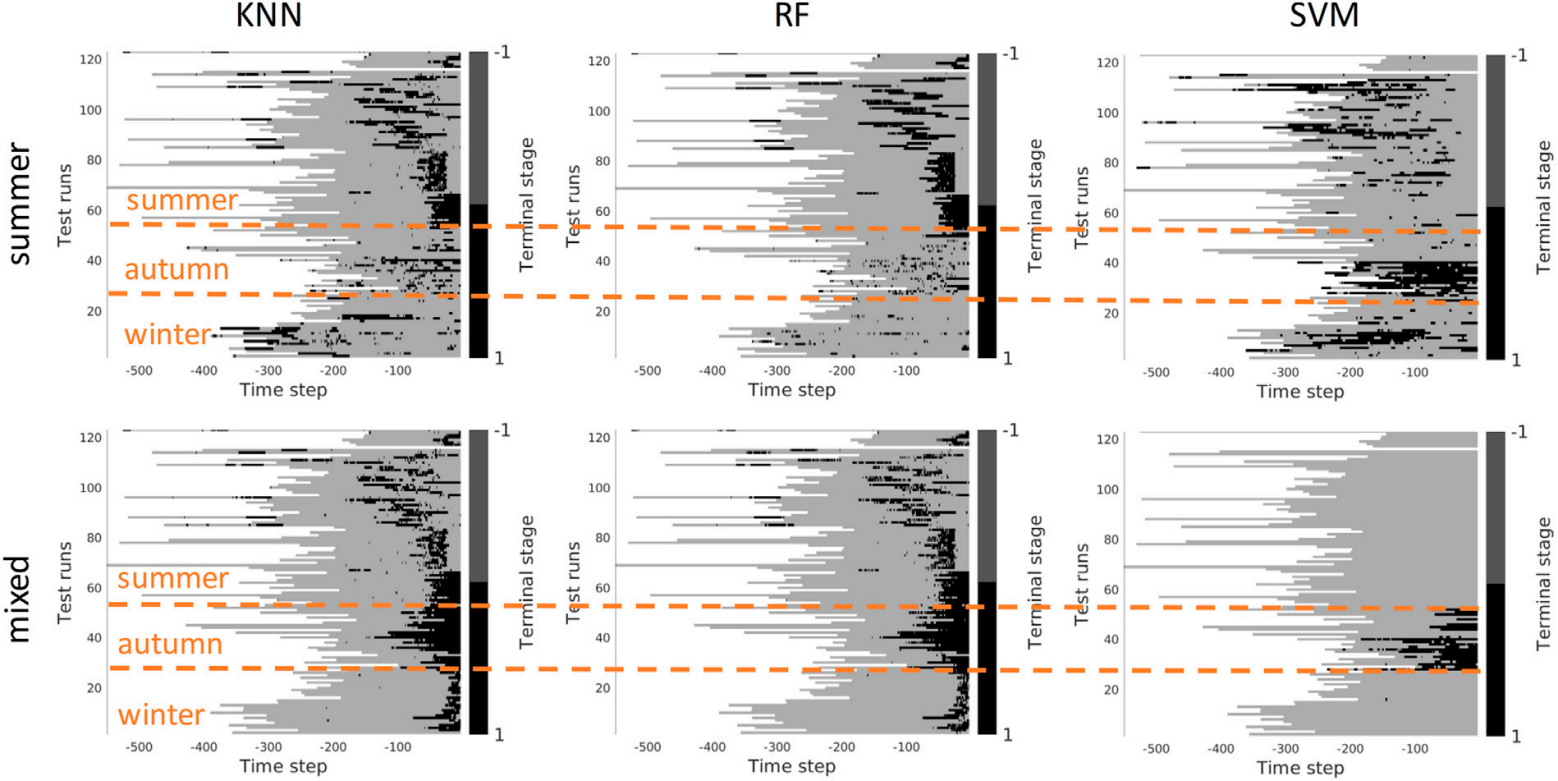
Identification of the terminal stage plotted for all seasons. Upper row: scenario 1 - trained on the summer set of data *D*_*summer*_). Lower row: Scenario 2 - trained on the mixed set of data. *x*-axis stands for the time step in reversed order to match the samples of varying length. Color bar encodes whether the machine reached terminal stage or not: non-terminal - gray, terminal - black.

Figure 13 demonstrates the results of the terminal stage prediction. With these experiments, we intended to verify whether the task-specific contrast features (trained for vehicle control) can be used to detect the terminal stage of the task. When all seasons are mixed the results look good and provide a high classification rate. However, when only trained on summer and tested on the rest of the seasons, the results are not satisfactory. In this case, the contrastive part of the loss seems to allow us to learn the invariant part of the visible aspects of the task. The results of terminal stage classification are more solid compared to stage classification also because it was easier for the expert to label the terminal stage of the task - when the task is completed.

## 5 Conclusion

We explored the stage-based reward prediction from a visual observation implemented for a real-world long-horizon robotic task. In our set-up, a wheel-loader performs a pile loading task. The reward is predicted from visual observations. We experimented with several most common visual representations used for imitation and reinforcement learning. In prior work, both the stage-based reward and visual representations were tested in laboratory conditions. Here we report the results for the outdoor pile loading task performed during three seasons.

Our question was whether the visual features and reward prediction models can be transferred between seasons. The results suggest that neither of the commonly used visual representation allows transfer from summer to other seasons without a loss of performance. The smallest drop of accuracy was produced with depth features. The best performance was achieved by mixing the data from all seasons. In this case, the most reliable results are achieved with depth and deep pre-selected VGG features. Time-contrastive features seem not to be efficient when trained from scratch on a small set of data. They seem to be less effective when the visual data does not contain a third-person view reflecting the structure of motion. As for real-world implementation, one should consider how the training data is labeled and which combinations of feature/classifier are computationally feasible to use.

In the future, we plan to investigate the problem of automatic ground truth generation, for example, by retrospective sensory data. This problem seems to be the bottleneck for the majority of current industries except autonomous driving in urban environments where an abundance of data is available. We will also study the methods to associate the visual observation with a map of the observed location rather than just one reward number. We will explore what form of this cost map is most suitable for action planning.

## Data Availability

The raw data supporting the conclusions of this article will be made available by authors upon a request.

## Footnotes

1
https://scikit-image.org/docs/dev/auto_examples/features_detection/plot_hog.html

2
https://github.com/nianticlabs/monodepth2

3
https://scikit-learn.org/stable/modules/svm.html

4 Avant 635 is a multi-purpose loader and also used for pallet loading, thus the boom (manipulator) comes with an extra degree of freedom a telescopic (prismatic) boom, which is not used in this study since it is not common in earth moving.
